# Pretreatment Factors Influencing Radiation Pneumonitis after Stereotactic Body Radiation Therapy in the Treatment of Lung Cancer

**DOI:** 10.7759/cureus.7462

**Published:** 2020-03-29

**Authors:** Alexander A Harris, Kyle Stang, Christina Small, Ryan Hutten, Fiori Alite, Bahman Emami, Matthew Harkenrider

**Affiliations:** 1 Radiation Oncology, Loyola University Chicago Stritch School of Medicine, Maywood, USA; 2 Radiation Oncology, Huntsman Cancer Hospital, University of Utah, Salt Lake City, USA; 3 Radiation Oncology, Geisinger Cancer Center, Danville, USA

**Keywords:** stereotactic body radiation therapy, sbrt, radiation pneumonitis, pretreatment factors, lung cancer, sabr, treatment related toxicity, stereotactic ablative radiation

## Abstract

Objective

Radiation pneumonitis (RP) is a dose-limiting toxicity that affects the treatment of lung cancer. Data on factors predictive of developing symptomatic RP after stereotactic body radiation therapy (SBRT) are limited. We reviewed data to identify pretreatment factors predictive of the development of symptomatic RP in patients’ lung cancer treated with SBRT.

Methods

Data were collected on 296 patients treated with SBRT for lung cancer. Factors available at time of consultation were analyzed for the development of symptomatic RP, defined as CTCAE v. 4.0 ≥ Grade 2. The factors analyzed included patient demographic, tumor-specific, and pretreatment pulmonary function data. Univariate and multivariate analyses were performed to assess for predictive factors.

Results

Median follow-up was 22 months. The rate of symptomatic RP was 16%. Univariate analysis showed an increased rate of symptomatic RP with treatments to the right lung (22% vs. 9%, p = 0.007), driven primarily by an increased rate of symptomatic RP when treating the right lower lobe (RLL) vs. other lobes (31 vs. 13%, p = 0.03). Patients with a history of prior lung directed therapy were also more likely to develop symptomatic RP (12% vs. 24%, p = 0.008). These statistical differences were retained on multivariate analysis.

Conclusion

SBRT to the right lung, especially the RLL, and to patients with a history of prior lung-directed therapy increases the risk of developing symptomatic RP after SBRT. Further studies on ways to predict and prevent symptomatic RP are needed.

## Introduction

Lung cancer is the leading cause of cancer death in the United States with an estimated 234,030 new cases of lung cancer and 154,050 deaths from lung cancer in 2018 [1]. Radiation therapy has a well-established role in treating patients with locally advanced lung cancer. The role of radiation therapy in early stage lung cancer is evolving. Early studies using standard fractioned radiation therapy for early stage lung cancer showed poor local control rates and high rates of treatment-related toxicity, specifically radiation pneumonitis (RP) [2-4]. Recent studies, using more modern radiation techniques with high dose per fraction treatment radiation therapy via stereotactic body radiation therapy (SBRT) have shown excellent local control with low rates of clinically relevant RP [4-6]. This has led to use of SBRT in treating patients with early stage lung cancer that have been deemed medically inoperable or in those who decline definitive surgery. Studies are now assessing the role of SBRT in treating medically operable patients [7, 8].

The primary toxicity associated with radiation therapy to the lungs is RP, which is the result of injury to type II pneumocytes within the lung, resulting in impaired surfactant production and damaged alveolar structure. As a result, an inflammatory response ensues and can compromise gas exchange and decrease lung compliance, leading to a decrease in pulmonary function. Patients that develop symptoms of radiation pneumonitis often present with dyspnea, cough, and fever. The mainstay of managing mild, acute symptomatic RP is corticosteroids. More severe cases may require supplemental oxygen or mechanical ventilation [9].

Factors predictive of symptomatic RP have been described for many modalities of radiotherapy. Pretreatment patient factors including patient age, cancer location, smoking status, chemotherapy schedule and medical comorbidities have been identified as clinical risk factors for the development of symptomatic RP in patients treated with conventionally fractionated radiation therapy [10]. In patients treated with SBRT, dosimetric factors including gross tumor volume, planning target volume, total volume of lung receiving 5 Gy (TLV5), 20 Gy (TLV20), contralateral V5, and mean lung dose (MLD) were found to be associated with the development of symptomatic RP [6, 11]. However, there is limited data evaluating pretreatment, patient-specific factors that influence the development of RP in patients receiving SBRT. These patient-specific factors could be useful in explaining the pros and cons of SBRT as compared with other treatments to a patient when they are evaluating which treatment modality they wish to pursue. The aim of this study is to evaluate patient-specific, pretreatment factors available at time of consultation that influence the rate of RP in patients treated with SBRT for primary or secondary lung cancer.

## Materials and methods

After approval from the institutional review boards, we reviewed 296 patients who received SBRT for either primary or secondary lung cancers. Patients were treated between 2005 and 2015. Initial staging was performed with computer tomography (CT) scans and routinely with 18F-deoxyglucose positron emission tomography scan. All patients were treated at Loyola University Medical Center or Edward Hines Jr. VA Hospital with linear accelerator-based SBRT. Our routine simulation process performed throughout the study period was a 3D CT planning scan followed by 4D CT scan through the region of the lesion in the treatment position in an immobilization device. Abdominal compression was utilized when breathing motion was determined to be clinically significant at the discretion of the treating physician. Contouring was completed on free breathing 3D CT and maximum intensity projection (MIP) image sets from the 4D CT to create the internal target volume (ITV). The planning target volume (PTV) was defined as ITV with a uniform 5-mm margin. SBRT was delivered with non-coplanar conventional 3D beams or with volumetric modulated arc therapy (VMAT). The patients were treated to a dose of 50-60 Gy prescribed to the PTV in three to five fractions using stereotactic body radiation, with central lesions receiving 50 Gy in five fractions, and peripheral lesions receiving 54 Gy in three fractions or 60 Gy in five fractions.

Patient demographic, tumor-specific, and pulmonary function test (PFT) data that was available at the time of initial consultation were assessed for a relationship with the development of symptomatic RP. The specific patient demographic, tumor-specific, and pretreatment PFT data analyzed are listed in Table 1.

**Table 1:** List of Pretreatment Patient Demographic, Tumor-Specific, and PFT Data PFT: Pulmonary Function Test; KPS: Karnofsky Performance Score; FVC: Forced Vital Capacity; FVC % Predicted: FVC Forced Vital Capacity Percent Predicted; FEV1: Forced Expiratory Volume in One Second; FEV1 % Predicted: Forced Expiratory Volume in One Second Percent Predicted; DLCO: Diffusion Capacity of the Lung for Carbon Monoxide; DLCO % Predicted: Diffusion Capacity of the Lung for Carbon Monoxide Percent Predicted.

Patient Demographic Data	Tumor-Specific Data	Pretreatment PFT Data
Age	Histology	FVC
Gender	Laterality (left vs. right)	FVC % Predicted
KPS at Time of Treatment	Axial Location (central or peripheral)	FEV1
Smoking Status	Upper/Lower (middle grouped with lower)	FEV1 % Predicted
Pack Years Smoked	Lobe Treated	FEV1/FVC
Prior Lung Directed Therapy (prior radiation or surgery)	T-Stage	DLCO
	Number of Sites Treated (single or multiple)	DLCO % Predicted

RP was assessed by the practicing physician at the time of treatment and at interval follow-up visits performed every 3-6 months for years 1-2, every 6-12 months for years 3-5, and then annually. RP was graded according to the Common Terminology Criteria for Adverse Events, version 4.0 (CTCAE v. 4.0). Symptomatic RP was defined as CTCAE v. 4.0 grade 2 or greater [12].

Statistical analysis

Descriptive statistics were calculated for categorical variables (frequency and percentages) and for continuous variables (mean with standard deviations and medians with ranges). Patients were dichotomized into two groups according to the maximum reported treatment related pneumonitis grade that they experienced. The two groups were asymptomatic patients (grade 0 or 1 pneumonitis) and symptomatic patients (grade 2 or greater). Categorical pretreatment factor variables were tested for independence using Fisher’s Exact tests and Chi-Squared tests. Continuous pretreatment factor variables were tested for correlation using univariate logistic regression using Spearman correlation coefficients. A binary logistic regression model was utilized to perform a multivariate analysis. Any variable with p ≤ 0.20 on univariate analysis was included in the multivariate analysis. Two multivariate analyses were performed to account for the interdependence of lobe location with lung laterality and upper/lower lobe location. The software system SPSS (IBM SPSS Statistics for Windows, version 25.0; IBM Corp., Armonk, NY, USA) was used for data analysis.

## Results

The results from 296 patients’ records were analyzed. The median follow-up was 22 months (range 1-116 months). Patient demographic, tumor-specific, and pretreatment PFT data are listed in Table 2. Of the 296 patients treated, 48 (16%) developed symptomatic RP. There was one case of grade 3 pneumonitis and one case of grade 4 pneumonitis. There were no cases of grade 5 RP in the data series.

**Table 2:** Demographic, Tumor-Specific, and PFT Data of All Patients For continuous variables, the means with standard deviations (SD) in parentheses are outlined as are the medians with ranges in parentheses. For categorical variables, the numbers with corresponding percentages in parentheses are outlined. PFT: Pulmonary Function Test; KPS: Karnofsky Performance Score; FVC: Forced Vital Capacity; FVC % Predicted: FVC Forced Vital Capacity Percent Predicted; FEV1: Forced Expiratory Volume in One Second; FEV1 % Predicted: Forced Expiratory Volume in One Second Percent Predicted; DLCO: Diffusion Capacity of the Lung for Carbon Monoxide; DLCO % Predicted: Diffusion Capacity of the Lung for Carbon Monoxide Percent Predicted; LUL: Left Upper Lobe; LLL: Left Lower Lobe; RUL: Right Upper Lobe; RML: Right Middle Lobe; RLL: Right Lower Lobe; NSCLC: Non-Small-Cell Lung Carcinoma; SCLC: Small Cell Lung Carcinoma.

Patient Demographic Data		Tumor-Specific Data		Pretreatment PFT Data	
Age		Laterality		FVC	
Mean (SD)	71 (10.5)	Left	120 (48%)	Mean (SD)	2.69 (0.91)
Median (Range)	72 (22-94)	Right	130 (52%)	Median (Range)	2.49 (0.95-5.4)
Gender		Axial Location		FVC % Predicted	
Male	119 (52%)	Central	68 (31%)	Mean (SD)	82.1% (22.9)
Female	109 (48%)	Peripheral	150 (69%)	Median (Range)	80% (30-150%)
KPS		Upper/Lower		FEV1	
100	17 (6%)	Upper Lobes	175 (70%)	Mean (SD)	1.64 (0.71)
90	92 (35%)	Lower Lobes	75 (30%)	Median (Range)	1.50 (0.45-4.05)
80	90 (34%)	Lobe Treated		FEV1 % Predicted	
70	46 (17%)	LUL	84 (34%)	Mean (SD)	64.1% (24.1)
60	14 (5%)	LLL	36 (14%)	Median (Range)	62% (15-138%)
50	4 (2%)	RUL	76 (30%)	FEV1/FVC	
Smoking Status		RML	15 (6%)	Mean (SD)	61.2% (13.8)
Current	68 (23%)	RLL	39 (16%)	Median (Range)	63% (26-95%)
Former	201 (68%)	Histology		DLCO	
Never	27 (9%)	Metastatic	36 (14%)	Mean (SD)	12.1 (4.95)
Pack Year Smoked		NSCLC	222 (84%)	Median (Range)	11.4 (2.33-28.5)
Mean (SD)	50.2 (36.7)	SCLC	5 (2%)	DLCO% Predicted	
Median (Range)	45 (0-180)	T-Stage		Mean (SD)	55.1% (22)
Prior Lung Treatment		T1	184 (72%)	Median (Range)	54.5% (9-117%)
Yes	72 (35%)	T2	60 (23%)		
No	133 (65%)	T3	12 (5%)		
		Number of Sites			
		Single	258 (87%)		
		Multiple	38 (13%)		

Comparing patient demographic data

In comparing patients that went on to develop RP and the patients that were asymptomatic from SBRT treatment, there were no statistically significant differences in age, gender, KPS at time of treatment, smoking status, or in the average pack years smoked. Patients that had previously undergone lung-directed therapy (prior thoracic surgery or radiation therapy) were found to have an increased risk of developing symptomatic radiation pneumonitis as compared to those who had no history of prior lung therapy (21% vs. 15%, p = 0.008). These findings are outlined in Table 3.

**Table 3:** Comparison of Patient Demographic Data Between Asymptomatic and Symptomatic Patients For continuous variables, the means with standard deviations in parentheses are outlined. For categorical variables, the numbers with percentages in parentheses are outlined. KPS: Karnofsky Performance Score

	Asymptomatic	Symptomatic	p-value
Age:	71.4 (10.11)	69.3 (12.4)	0.223
Pack-Years Smoked:	50.1 (37.4)	50.6 (33.4)	0.948
Gender:			0.584
Male	150 (85%)	27 (15%)	
Female	98 (82%)	21 (18%)	
KPS at Time of Treatment:			0.645
100	12 (71%)	5 (29%)	
90	79 (86%)	13 (14%)	
80	74 (82%)	16 (18%)	
70	38 (83%)	8 (17%)	
60	12 (86%)	2 (14%)	
50	4 (100%)	0 (0%)	
Smoking Status:			0.146
Current	62 (91%)	6 (9%)	
Former	163 (81%)	38 (19%)	
Never	23 (85%)	4 (15%)	
Prior Lung Directed Therapy:			0.008
Yes	57 (79%)	15 (21%)	
No	113 (85%)	20 (15%)	

Tumor-specific results

In comparing rates of symptomatic RP, there were no differences across axial location, upper/lower lobe location, histological type, T-stage, or if multiple sites were treated at one site. However, there was a statistically significant higher rate of symptomatic RP for patients that were treated to the right lung over the left lung (22% vs. 9%, p = 0.007). The highest rate of symptomatic RP was seen in the right lower lobe (RLL) with a rate 31% (p = 0.03). These significant differences were observed in both univariate and multivariate analysis for both the lung laterality (p = 0.007 and p = 0.004) and the specific lobe treated (p = 0.03 and p = 0.001). These findings are outlined in Table 4.

**Table 4:** Comparison of Tumor-Specific Data Between Asymptomatic and Symptomatic Patients The numbers with corresponding percentages in parentheses are outlined. LUL: Left Upper Lobe; LLL: Left Lower Lobe; RUL: Right Upper Lobe; RML: Right Middle Lobe; RLL: Right Lower Lobe; NSCLC: Non-Small-Cell Lung Carcinoma.

	Asymptomatic	Symptomatic	p-value
Axial Location			0.486
Central	55 (81%)	13 (19%)	
Peripheral	127 (85%)	23 (15%)	
Upper/Lower Lobes			0.102
Upper Lobes	152 (87%)	23 (13%)	
Lower Lobes	59 (79%)	16 (21%)	
Laterality			0.007
Left Lung	109 (91%)	11 (9%)	
Right Lung	102 (78%)	28 (22%)	
Lobe Treated			0.03
LUL	77 (92%)	7 (8%)	
LLL	32 (89%)	4 (11%)	
RUL	63 (83%)	13 (17%)	
RML	12 (80%)	3 (20%)	
RLL	27 (69%)	12 (31%)	
Histology			0.39
Metastatic	29 (81%)	7 (19%)	
NSCLC	189 (85%)	33 (15%)	
Small Cell	3 (60%)	2 (40%)	
T-Stage			0.115
T1	156 (85%)	28 (15%)	
T2	44 (73%)	16 (27%)	
T3	9 (75%)	3 (25%)	
Multiple Sites Treated			0.23
No	221 (86%)	37 (14%)	
Yes	27 (71%)	11 (29%)	

Pretreatment PFT results

In comparing the pretreatment FVC, percent predictive FVC, FEV1, percent predicted FEV1, FEV1/FVC, DLCO and the percent predictive diffusion limit of carbon monoxide, there were no significant differences between patients that had developed symptomatic RP and those that did not. These findings are outlined in Table 5.

**Table 5:** Comparison of Pretreatment PFT Data Between Asymptomatic and Symptomatic Patients The means with standard deviations (SD) in parentheses and the medians with the ranges in parentheses are outlined. PFT: Pulmonary Function Test; FVC: Forced Vital Capacity; FVC % Predicted: FVC Forced Vital Capacity Percent Predicted; FEV1: Forced Expiratory Volume in One Second; FEV1 % Predicted: Forced Expiratory Volume in One Second Percent Predicted; DLCO: Diffusion Capacity of the Lung for Carbon Monoxide; DLCO % Predicted: Diffusion Capacity of the Lung for Carbon Monoxide Percent Predicted.

	Asymptomatic	Symptomatic	p-value
FVC			0.829
Mean (SD)	2.68 (0.94)	2.72 (0.76)	
Median (Range)	2.48 (0.95-5.4)	2.58 (1.33-4.93)	
FVC % Predicted			0.738
Mean (SD)	80.9% (25)	82.7% (20.4)	
Median (Range)	78% (30-150%)	83.5% (45-115%)	
FEV1			0.649
Mean (SD)	1.65 (0.72)	1.58 (0.65)	
Median (Range)	1.50 (0.45-4.05)	1.53 (0.54-3.01)	
FEV1 % Predicted			0.664
Mean (SD)	62.9% (25)	65.2 (23.9)	
Median (Range)	62% (15-138%)	66% (23-105%)	
FEV1/FVC			0.738
Mean (SD)	60.2% (15.9)	61.3 (13.0)	
Median (Range)	63% (26-95%)	63.5 (32-82%)	
DLCO			0.536
Mean (SD)	12.0 (5.1)	12.7 (4.2)	
Median (Range)	11.4 (2.33-28.5)	12.6 (3.7-19.3)	
DLCO % Predicted			0.833
Mean (SD)	55.2% (21.5)	54.3% (24.5)	
Median (Range)	54.0% (10-117%)	55.0% (9-110%)	

Multivariate analysis

Upper/lower lobe, lung laterality, lobe treated, tumor stage, and prior lung treatment were the variables identified through univariate analysis to be assessed in the multivariate analysis. Two separate multivariate analyses were performed in order to account for the interdependence between lobe location with upper/lower lobe and lung laterality. Lung laterality, lobe treated, and prior lung treatment remained statistically significant on multivariant analysis. Results are shown in Table 6.

**Table 6:** Multivariate Analysis with Corresponding p-values UVA: Univariate Analysis; MVA: Multivariate Analysis.

	UVA p-value	MVA p-value
Upper/Lower Lobes	0.102	0.217
Lung Laterality	0.007	0.016
Lobe Treated	0.03	0.006
T-Stage	0.115	0.031
Prior Lung Treatment	0.008	0.012

## Discussion

RP is a relatively common yet troublesome toxicity in patients treated with radiation for lung cancer. The rate of developing symptomatic RP in our study sample was 16%, which is similar to other published rates for patients treated with SBRT [11-16]. Our study also identified that SBRT treatment in patients with a history of prior lung-directed therapy and treatment to the right lung, specifically the RLL, are at greater risk of developing symptomatic RP. The ability to evaluate and appropriately counsel patients on their individualized risk of developing radiation pneumonitis is important to treating radiation oncologists.

There has been significant research on both patient-specific and dosimetric data that correlate with an increased risk of patients developing RP for standard fractionation radiation therapy. A literature-based meta-analysis by Vogelius and Bentzen reviewed 31 studies that assessed various patient-specific factors to assess for correlations in the development of RP [10]. The authors found that older age, presence of pulmonary comorbidity and tumor location in the middle or inferior part of the lung increased the rate of developing symptomatic RP in patients treated with conventionally fractionated radiation therapy. In contrast, they found that a current smoker or former smoker status was protective and decreased the rate of symptomatic RP. Our data did not replicate the influence of age and smoking status on symptomatic RP in the setting of SBRT. However, we noted the highest rates of symptomatic RP in our sample were in patients treated specifying to the RLL.

There has been a number of studies evaluating dosimetric data that correlates with the risk of symptomatic RP. Nakamura et al. reviewed data patients treated with Cyberknife-based SBRT for lung cancer [11]. They assessed the dosimetric data of 56 patients to find potential correlations with symptomatic RP. In their univariate analysis, they found that maximum tumor diameter, gross tumor volume, planning target volume, mean lung dose, and a normal lung volume receiving 5-50 Gy of radiation (V5-V50) were significant predictors for symptomatic RP. However, in the multivariate analysis, only a V25 > 3.4% was identified as a significant predictive factor of symptomatic RP. Other studies have further evaluated dosimetric parameters for their risk of developing symptomatic RP and found associations with a number of factors including GTV size, PTV size, lung V5, lung V10, lung V20, and ipsilateral mean lung dose [14-16]. These variables associated with symptomatic RP derive from the later phase of treatment planning, after the majority of treating radiation oncologists have already outlined the risks and benefits to the patient at time of consultation. When these dosimetric variables would be discovered, the patient would have already agreed to SBRT treatment. Pretreatment, patient-specific factors available at the time of consultation could provide added insight for patients and treating physicians as to if SBRT is the optimal choice for treatment.

Baker et al. looked to evaluate these pretreatment, patient-specific factors along with dosimetric variables to search for their potential impact on symptomatic RP [17]. On univariate analysis, they found female gender, performance status, V5, V13 and V prescription, and planning target volume to normal lung volume ratio were significantly predictive of RP. However, on multivariate analysis, only female gender, pack-years smoking, and larger gross ITVs and PTVs were predictive of symptomatic RP.

The aim of our study was to assess for a number of patient-specific factors that were available at the time of consultation to assess for potential risk for symptomatic RP, with the hopes of more accurately educate patients and other medical practitioners of an individual’s risk of developing symptomatic RP. Dosimetric data was not evaluated in this study, as we limited our review to factors available at the time of initial consultation. We discovered that patients who have already received lung-directed therapy as well as those who are planned to receive treatment to the right lung, more specifically the RLL, were more likely to develop symptomatic RP. On multivariate analysis, we also found that patients with a higher tumor stage (T2 or T3) were at greater risk for symptomatic RP. The result from the study of Baker et al. showing gender and smoking history having an impact on rates of symptomatic RP was not replicated in our study [17].

The exact cause of the increased risk of symptomatic RP with treatment of the right lung is still unknown. We hypothesize that it may be due to the greater volume of the right lung relative to the left lung. Treatment to the right lung likely results in a greater volume of normal lung being irradiated, and thus an increased risk of RP. This phenomenon is also seen in surgical data that showed increased lethality in patients treated with right-sided pneumonectomy vs left-sided pneumonectomy [18, 19]. Furthermore, lesions in the lower lobes tend to move more during the respiration cycle. Because of this, treating radiation oncologists are often forced to treat greater volumes of normal lung in order to ensure adequate tumor coverage. Additionally, the majority of patients treated have an extensive smoking history, and associated obstructive lung disease, which has a higher predilection for damaging the upper lung lobes. This results in the lower lobes making up a higher proportion of the functional lung volume in these patients. These reasons likely explain why we saw the greatest rates of symptomatic radiation pneumonitis in patients treated to the RLL.

Our data also showed that the relative risk of developing symptomatic RP increased by about 40% in patients that had already undergone lung-directed therapy. Other studies have reported similar findings with patients who received prior radiation or prior surgery [20-22]. These studies closely examined not only toxicity rates of patients with a history of prior surgery or radiation therapy, but also control and survival rates associated with their treatment. These reports showed high rates of control with acceptable rates of toxicity, as did our results [17-19]. The cause of increased toxicity in treating these patients likely corresponds to the fact that a greater proportion of their remaining viable lung is being treated as compared to patients without lung-directed therapy. A correlation between the ratio of treated lung volume to untreated lung volume and the increased risk of pneumonitis was not directly assessed in our study, but has been reported previously [11,13-17].

The single institution nature of our study design led to a relatively small population treated by a small number of practitioners. With a larger study consisting of multiple treating intuitions, or the pooling of data in a meta-analysis, it may be possible to identify additional factors that may influence RP, as well as make the results more generalizable to a larger patient population. Additionally, the retrospective design of the study led to the utilization of some incomplete patient data. Aspects of patient data that were missing were omitted from our calculations. This led to a decrease in the amount of analyzable data for specific pretreatment factors. With a prospectively collected study, the complete data would likely be available for each and every pretreatment factor and thus would also increase the overall amount of analyzable data. Future studies should work to assess the impact of pretreatment factors on the potential for developing RP in a multi-institutional, prospectively collected manner.

## Conclusions

In this study, we retrospectively reviewed data to assess for correlations between patient-specific pretreatment factors and the development of symptomatic RP. Our dataset showed an overall symptomatic RP rate of 16%. We found that SBRT to the right lung, especially the RLL, and treatment of patients with a history of prior lung-directed therapy increased the rate of symptomatic RP. These findings and additional research in this area will allow for radiation oncologists to more accurately evaluate and counsel patients on their individualized risk of potentially developing RP prior to receiving SBRT treatment, and more confidently diagnose and treat RP after treatment with SBRT.
